# Training a high accuracy model to visualize blood clots during mechanical thrombectomy for the treatment of Acute Ischemic Stroke

**DOI:** 10.3389/fstro.2025.1610399

**Published:** 2025-10-17

**Authors:** Varun Kashyap, Richard Zhu, Karthik Narasimhan

**Affiliations:** ^1^Research and Technologies, Medtronic Neurovascular, Irvine, CA, United States; ^2^Department of Computer Science, Princeton University, Princeton, NJ, United States

**Keywords:** Acute Ischemic Stroke, mechanical thrombectomy (MT), stent retriever (SR), clot visualization, machine learning (ML), computer vision, image segmentation-deep learning, artificial intelligence (AI)

## Abstract

**Background:**

Mechanical thrombectomy is the standard of care for Acute Ischemic Stroke caused by proximal large-vessel occlusion in the anterior circulation. In the stent retriever approach, a nitinol stent engages the clot via outward radial force to enable removal. However, current procedures lack direct clot visualization under fluoroscopy, which can reduce retrieval efficacy and often require multiple passes. Improving first-pass success is critical given the time-sensitive nature of stroke intervention.

**Methods:**

This study presents a clot visualization method using the spatial arrangement of radio-opaque markers on the Medtronic Solitaire™ stent. A deep learning model, Clot[U]-Net, based on the U-Net architecture, was trained on 800 anteroposterior and lateral *in-vitro* images and evaluated on a separate test set.

**Results:**

The Clot[U]-Net model achieved strong performance in clot boundary prediction, with a mean Intersection over Union (IOU) of 87.9% and an AUROC of 89.9%, and standard deviations of 2.2 and 3.16, respectively.

**Conclusion:**

The proposed method enables clot visualization during stent retriever thrombectomy without altering existing clinical workflows. With further pre-clinical and clinical validation, this approach may support real-time decision-making and improve procedural outcomes.

## Introduction

**Figure 1:**
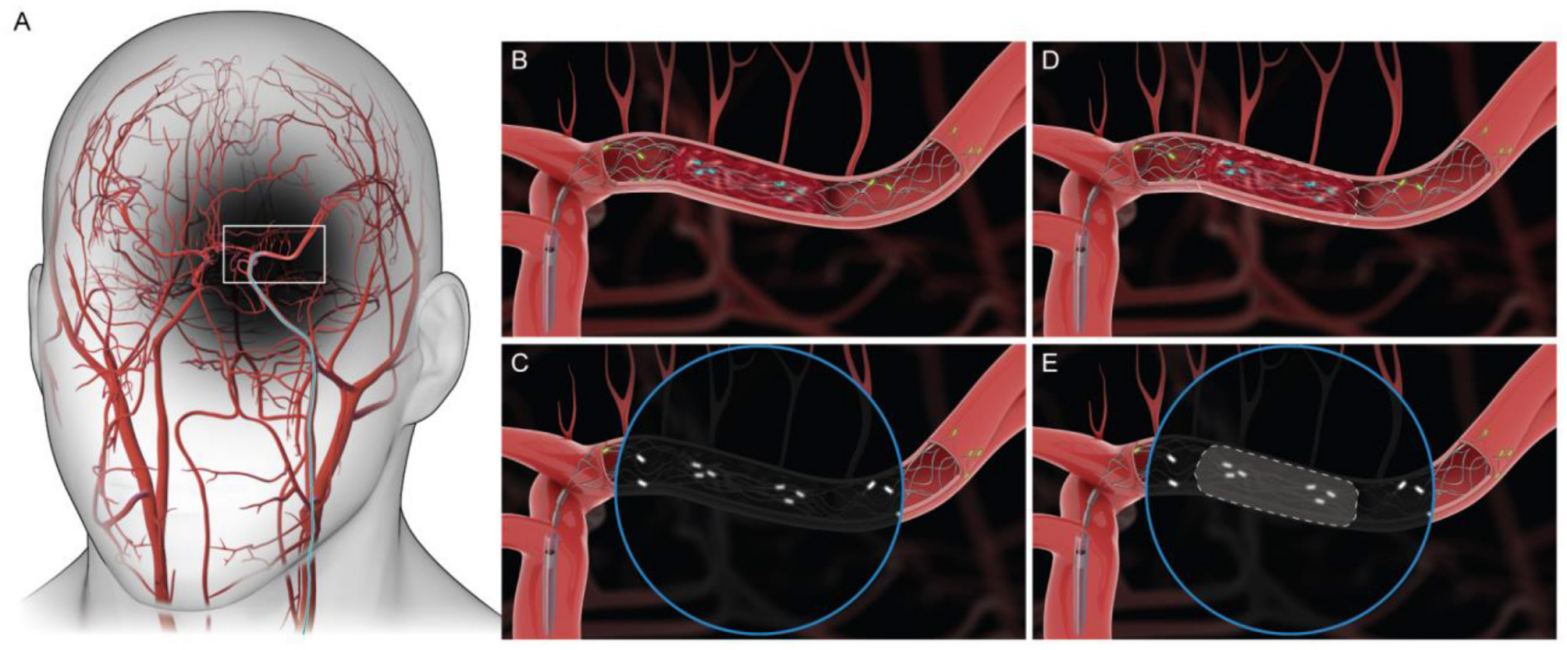
**(A)** Access to the middle cerebral artery (MCA) during MT. **(B, C)** Graphic of Medtronic Solitaire's engagement with the clot. Circular inset represents fluoroscopic visualization of stent engagement with the clot. **(D, E)** Boundary of the clot is reconstructed using a predictive algorithm.

Acute Ischemic Stroke (AIS) is a type of stroke where a blood clot occludes a vessel, causing decreased blood flow to the brain, resulting in damage to brain cells. It is the most common type of stroke and represents about 87% of all strokes (Benjamin et al., 2017). In selected patients with disabling AIS, intravenous thrombolysis within 4.5 h and mechanical thrombectomy (MT) within 24 h of symptom onset significantly improve functional outcomes (Mendelson and Prabhakaran, 2021; Lambrinos et al., 2016). AIS with major intracranial vessel occlusion is commonly caused due to cardioembolism or atherosclerosis related *in situ* stenosis/occlusion (Horie et al., 2016). MT is used for treatment of AIS with both these causes (Tsang et al., 2019; Deng et al., 2019). Predominantly, two procedures and their combinations have been used in MT including stent retriever and aspiration. With aspiration, the clot is suctioned out using a catheter endovascularly. While in the stent retriever technique, a nitinol stent mechanically engages with the clot due to its outward radial force and the clot can be retrieved through the vasculature via an interventional procedure. This is an interventional procedure carried out under fluoroscopy. As shown in Figure 1, the stent is deployed against the clot and the platinum radio opaque markers help visualize the position of the stent. However, it is important to note that the entire procedure is carried out without being able to visualize the clot. This is because the clot is not radio opaque and is invisible during imaging. Clinical fluoroscopic image of this endovascular procedure (including the stent with platinum radio opaque markers deployed against a blood clot) is shown in Ntoulias et al. (2023). Not knowing the boundary of the clot can lead to potential challenges during clot retrieval resulting in reduced clot retrieval efficacy (Lee, 2023). Currently, multiple clot retrieval cycles may be required for complete clot removal, both in the case of chronic stenosis with superimposed thrombosis where stenting is needed to maintain vessel patency and acute cardioembolic stroke. Multiple thrombectomy passes can cause vessel wall injury and increase bleeding risk (Mohamed et al., 2021). Several techniques are being tested for effective clot retrieval including combination therapy, and Solumbra technique (Arslanian et al., 2019; Yi et al., 2021), wherein the stent retriever is used in combination with other techniques such as aspiration to retrieve clot more efficiently. However, based on stent retrieval only and combination therapies, the first pass efficacy or the percentage of the times that the entire clot is retrieved in the first attempt can range between 35 and 55% (Liang et al., 2020; Requena et al., 2023). Improvements in first pass efficacy are extremely critical in stroke treatment since brain cells continue dying every second blood flow is not restored leading to long term disability (Saver, 2006). The clots that could potentially break off from the stent retriever remain unseen until a subsequent fluoroscopy run shows a corresponding occlusion to flow (Yeo et al., 2019). There is a need to visualize the radiolucent clot under fluoroscopy without disrupting the mechanical integrity of the clot so as to prevent clot fragmentation and resulting distal embolization (Pilgram-Pastor et al., 2021; Chueh et al., 2016). Furthermore, studying interaction of stent retriever with the clot could provide insight into clot composition, which might inform decisions regarding the retrieval technique used (Shin et al., 2022; Gurary et al., 2025; Aliena-Valero et al., 2021). Concepts in deep learning, and computer vision can be used to develop a predictive algorithm for object detection, and segmentation (Chen et al., 2022; Gupta et al., 2022). The accuracy of prediction could be improved with the introduction of larger datasets, thereby creating a tool that could effectively create a digital clot twin (Hoffmann et al., 2022; Figueroa et al., 2012; van Genderingen et al., 2024). Prior works in computer vision have attempted to use UNets for organ-level or disease segmentation tasks (Krithika et al., 2022). Others have used different neural network architectures (e.g., Transformer-based U-Net) (Cao et al., 2022) or more information-dense imaging methods such as optical coherence tomography (Qin et al., 2025) or ultrasound (Chen et al., 2024).

In this study, we propose using the relative position of the radio opaque platinum markers on the stent to visualize boundaries of the clot. Medtronic's Solitaire was utilized in this study since it is the most widely used stent retriever. We used a training dataset containing a total of 408 anteroposterior (AP) view and 408 lateral view images. The model was trained to predict the boundary of the clot in a test dataset and compared against the ground truth using established machine learning metrics including intersection over union (IOU) and area under the receiver operating characteristic curve (AUROC).

## Materials and methods

**Figure 2:**
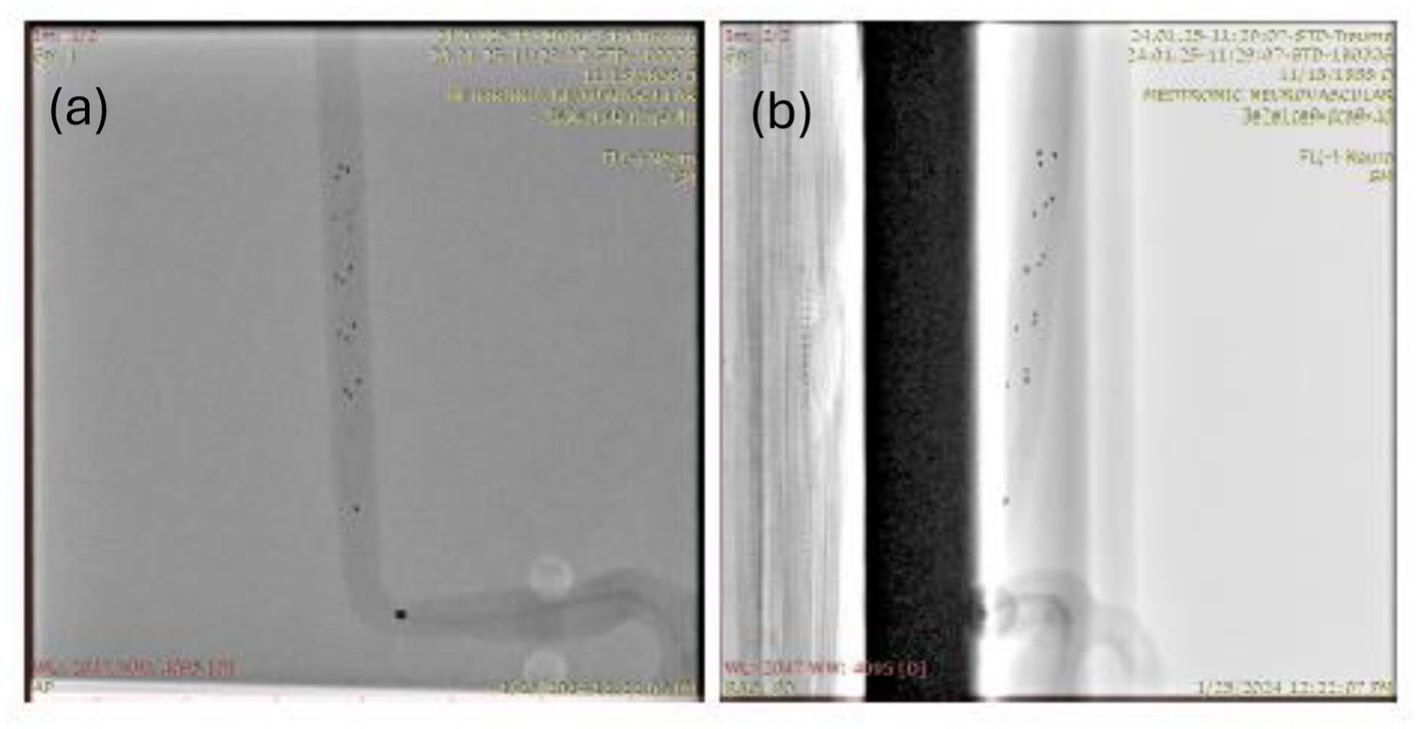
A sample input image of an *in-vitro* clot retrieval procedure with a Medtronic Solitaire Stent used as part of the test dataset shown in both AP and lateral views. **(a)** AP reference frame. **(b)** Lateral reference frame.

In this work, a computer vision model is developed to take fluoroscopic images as input (as shown in Figures 2a, b) and output a binary mask representing the location and shape of the clot. Since the boundary of the clot is not visible in the fluoroscopic image, the model picks up subtle deformations on the radiopaque markers to predict the location and boundary of the clot. The training dataset is used to train the model and tune the hyperparameters. Finally, a test data set is used to evaluate the performance and results are corroborated via visual comparison of generated clot predictions and optical images of the clots. Note that no ethical approval was needed for this work since the training data was obtained using an *in-vitro* model imaged under biplane fluoroscopy.

**Figure 3:**
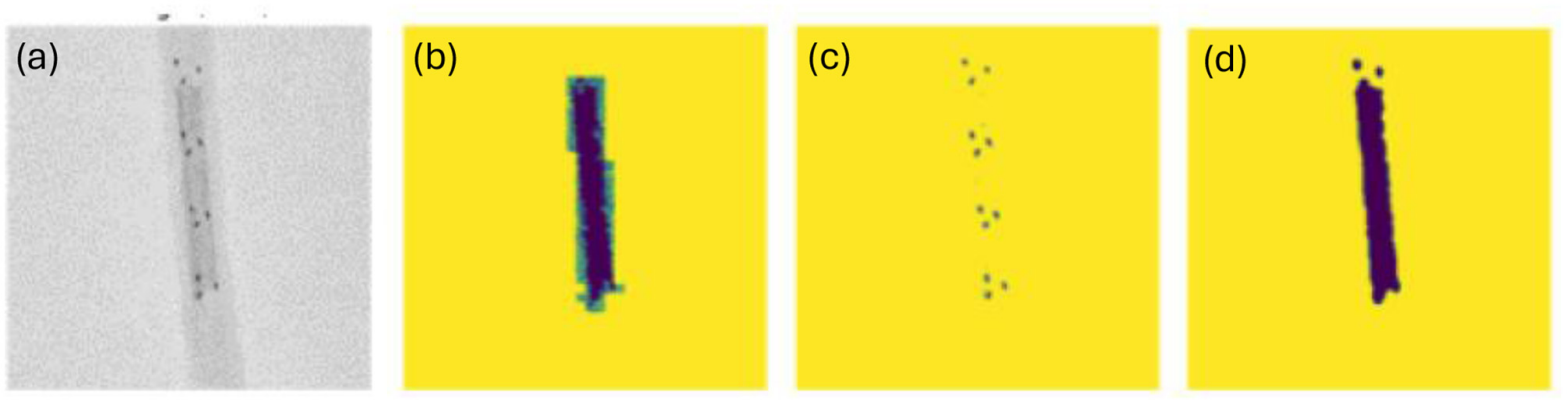
**(a)** Original image (a grayscale cropped version of the dataset); **(b)** Manually labeled mask representing the shape and location of the clot; **(c)** Result of using greyscale thresholding to extract shape and location of radiopaque markers; **(d)** Greyscale thresholding for clot.

**Figure 4:**
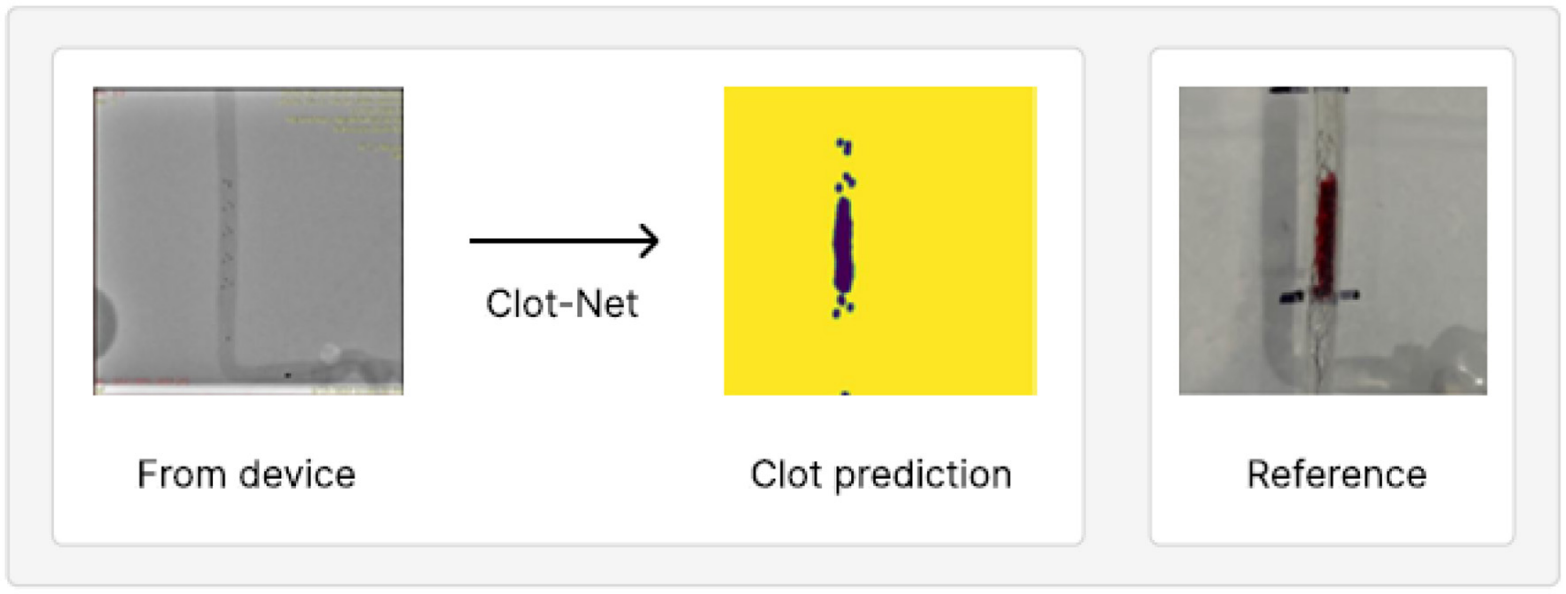
An example of model prediction robustness evaluated on our second, separately collected test set. Apart from the *in vitro* clot, the images are identical to those observed in clinical settings.

Dataset overview: The dataset comprises proprietary Medtronic fluoroscopic images produced specifically for this training and evaluation. It consists of 408 *in-vitro* Solitaire clot retrieval procedures imaged under fluoroscopy using a commercial C arm (Siemens-Healthineers Artis zee with PURE), in a biplane configuration. Each item in the dataset is a tuple of both an AP (top view) and lateral (side view) image produced by the two biplanar detectors. In the training dataset, the *in vitro* clot is prepared with a radiopaque compound (barium sulfate) for clot visualization as shown in Figure 3a. Note that this only for the purposes of obtaining the ground truth and is not possible during a clinical procedure. The clots used in this study were prepared using porcine blood, fibrinogen from bovine plasma and bovine plasma thrombin. The morphology of this clot maybe characterized to be between an RBC rich and fibrinogen rich clot. The clot was placed within a 2 mm internal diameter (ID) *in-vitro* vasculature. In the test split/dataset, the clots are not injected with barium sulfate, but instead a third optical image (as shown in Figure 4) of the clot is included in the tuple for manual verification of the approximate size and shape of the clot. Each image in the dataset has shape (908, 908, 3). The first two dimensions correspond to the height and width of the image in pixels, while the last dimension represents the 3 RGB channels.

Data processing: We use the Python programming language for data processing and developing the machine learning model. In order to prepare data for training and evaluation, we segmented the clot based on the AP view image. Typical 2-class image segmentation tasks use binary masks to represent each of the two classes. In this case, the two classes are the background and clot, of which the clot segmentation is of primary focus. Preprocessing primarily focuses on binarizing the data and removing timestamps and other text left on the image by the imaging system. We primarily use the *cv2, image*, and *numpy* Python libraries for data processing. The data was pre-processed by clot labeling and elimination of text annotations produced during fluoroscopic imaging that can be seen in Figures 2a, b.

Note that the images were taken in an environment controlled for contrast and brightness. This allowed us to first convert the images to grayscale, an action which had the added benefit of collapsing the last dimension of the image to a single channel. We then created a mask of the clot based on the areas of the image within a certain grayscale threshold. A visual side-by-side inspection of the radiopaque marker (Figure 3c) and clot masks (Figure 3d), with the respective ground-truth image (Figure 3a) across the training dataset confirmed high visual similarity. An additional potential challenge can be that a model trained on this training dataset containing radiopaque clots (as shown in Figure 2a) may struggle to generalize to the clinical environment where clots are not radiopaque. This is mitigated by using thresholding to generate a mask of the Solitaire markers/dots. We then train a model that predicts the clot mask from the dot mask. As shown in Figure 3, the threshold-based approach yields clot masks that are sometimes of even higher quality than the manually labeled clots, while also producing accurate dot masks.

Modeling: We propose Clot[U]-Net—a computer vision model based on the U-Net (Ronneberger et al., 2015), a popular architecture for segmentation tasks, with a novel training loss paradigm. A U-Net is, in essence, a machine learning model architecture that attempts to extract the most salient information from an image in iterative steps, before then reproducing a binary image that highlights the shape of the predicted clot. The training loss is a mathematical formula that dictates how the model should learn from training examples. Details of the U-Net architecture are provided in Supplementary material section of this manuscript. We train for 1,000 epochs and use a batch size of 32. An epoch represents one iteration of the model learning from the full training dataset and batch size is the number of individual examples the model looks at before updating its parameters. As opposed to parameters (of which the models have hundreds of thousands and learn automatically during training), hyperparameters are the architectural-level settings humans choose before training begins. In order to set the latter, we perform a rudimentary hyperparameter search by experimenting with various architectures and evaluating performance, as measured by both the visual and mean IOU/accuracy metrics. We choose the most effective hyperparametric configuration for our final model (more details on convolutional (e.g., number of filters, kernel size, stride length) and pooling layers are shown in Supplementary material of this manuscript). For evaluation, we use 2-class mean IOU, AUROC, and accuracy. We choose mean IOU since it is typically used when measuring segmentation model performance, models that identify shapes within images (Lin et al., 2024). The metric measures the number of overlapping pixels between predicted and actual clots relative to the union of prediction and actual. Since the clots are typically small relative to the total image area, this provides us a metric that aligns more closely with predictions that visually look good. We choose AUROC since it helps measure average classification ability at the pixel level compared to a random classifier. We secondarily refer to accuracy because it gives us a good baseline of what proportion of pixels are correctly predicted. Translation of this technique to the clinic is dependent on hardware requirements and latency due to post processing. While model training takes approximately 5–30 min using a state-of-the-art Nvidia H100 graphical processing unit (GPU), inference is possible in near real-time on a standard laptop GPU which can be integrated with current fluoroscopic systems.

Statistical methods: We further analyze the statistical significance of the mean IOU and AUROC scores for the test set. First, we assess whether the data is representative of a normal distribution using the Shapiro–Wilk test (α = 0.05), which suggests the data satisfies the normality assumption (IOU: W = 0.846, *p* = 0.183; AUROC: W = 0.949, *p* = 0.727). Proceeding with parametric analyses, we then compute for each metric the arithmetic mean (μ), sample standard deviation (s), standard error of the mean, and the 95 % confidence interval using the t-distribution with *n*−1 degrees of freedom:


$$\begin{array}{l} CI_{95\%}=\mu \pm t_{0.975,n-1}\times \frac{s}{\sqrt{n}}.\; \end{array}$$

## Results

Our model, based on a U-Net architecture, includes two differentiating aspects: 1. We use thresholding to separate image features before passing them into the U-Net (clot and dot masks described previously in data pre-processing paragraph) 2. During training, we alternate the loss function between binary cross entropy and mean IOU.

**Figure 5:**
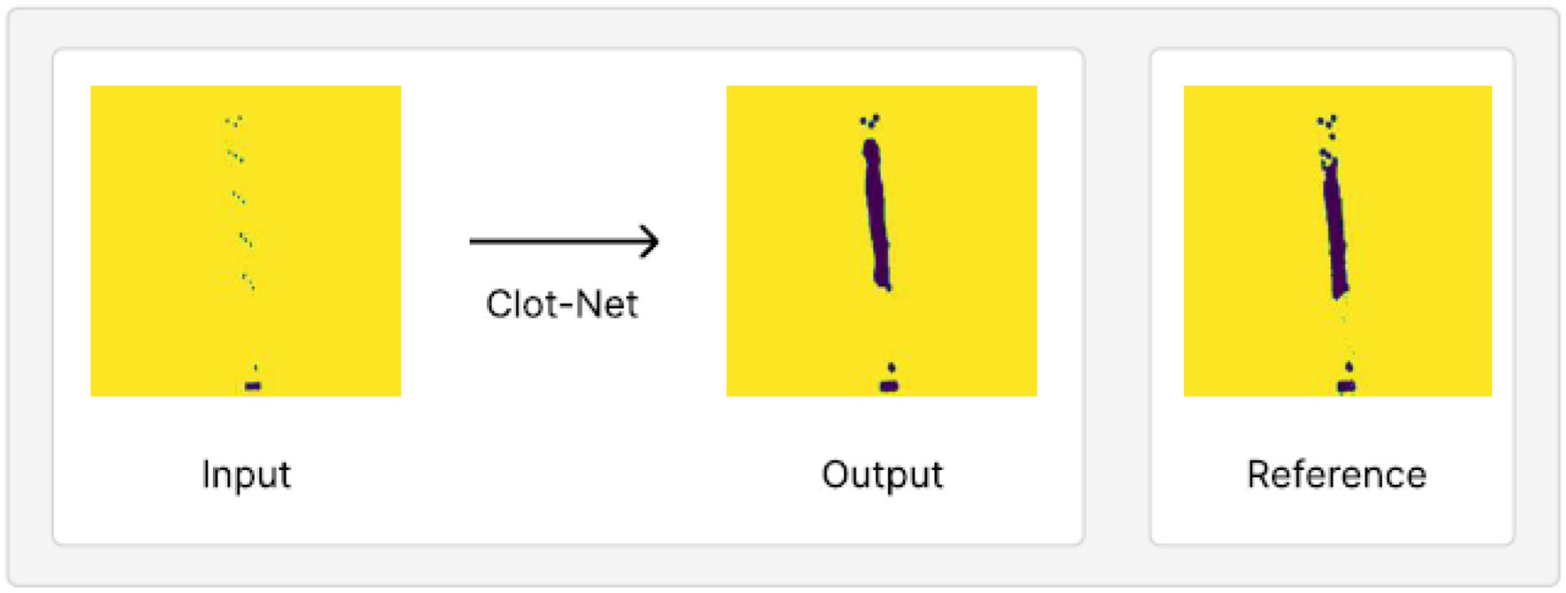
An example of model output on test set held out from our training/validation dataset, juxtaposed with the reference clot shape and location. This prediction achieves a mean IOU of 87.9% and AUROC of 89.9%.

We observe, qualitatively, that performance improves with separate addition of each of the above contributions relative to using the standard segmentation model paradigm. We also observe greater stability during training given our small dataset (the model consistently produces a well-trained model under our configuration as opposed to stochastic model prediction quality under the standard U-Net configuration). We evaluate ClotNet on a held-out test set (a dataset the model has not yet seen), producing reliable clot predictions (an instance of these is shown in Figure 5), and achieving an 85.8% mean IOU and an 89.9% AUROC under a 1,000 epoch configuration. Other observations during training and evaluation are described below. Additional model outputs are presented in Section 2 and Supplementary Figure S1. The input data contains low signal, by construction, since the dot mask used as input is 5 sets of 3 radiopaque markers. ClotNet, reliably approximated the boundary and position of the clot, typically performing well in the dimension normal to the clot. This is because the outlines of the predicted mask, and those of the reference clot, correspond closely with the left- and right-most markers as shown in the visual overlay of the input, output and reference images in Supplementary Figure S2, making it relatively easy to interpolate between clusters. However, lengthwise prediction accuracy appears to be relatively poor because of the distance between consecutive clusters of radiopaque markers. This limited information provided by markers could lead to ambiguity related to the position of distal end of the clot. Note that this model is trained with commercial clot retrieval stents. Clot prediction can be enhanced by future design changes to placement and number of radiopaque markers used on the stent.

ClotNet appears to be robust to distracting features, such as the radiopaque marker on the distal segment of the microcatheter (shown in Figure 5). It correctly passes this feature directly through to the output image, despite this microcatheter marker appearing in different locations across examples (shown in Supplementary Figures). This suggests that the model may be robust to distracting features, provided that they are seen in the training data, even when these features are larger than the stent markers themselves. The model also predicts accurately on a separately collected test dataset (see Figure 4 and Supplementary Figure S3) with different scaling, thereby further highlighting its promising performance for this application.

To evaluate the statistical significance of test set IOU and AUROC scores, we analyze the central tendency and dispersion of mean IOU and AUROC (see Table 1). Both metrics exhibit low variability across test images, based on the width of the confidence intervals.

**Table 1:** Mean, standard deviation, and confidence interval for test set metrics.

**Metric**	**Mean (μ)**	**Std. Dev. (*s*)**	**95% CI**
IOU (%)	85.78	2.20	[83.05, 88.51]
AUROC (%)	89.94	3.16	[86.02, 93.85]

The high average performance and narrow CIs confirm the model's robustness. Specifically, we note that our confidence interval width is near-best-in-class when compared with CI widths across 56 Medical Image Computing and Computer-Assisted Intervention segmentation challenges (Christodoulou et al., 2024).

## Discussion

In this work, we evaluate the feasibility of using a machine learning model to visualize boundary of the clot during MT. This is further confirmed using established metrics including IOU, AUROC and accuracy. With a limited dataset, the model is able to predict the boundary of the clot with mean IOU of 85.8% and AUROC of 89.9% and standard deviations of 2.2 and 3.2, respectively. Based on prior work in deep learning (Hoffmann et al., 2022; van Genderingen et al., 2024), it has been found that prediction accuracy can be improved with large training datasets. Model presented in this work can be further enhanced with larger datasets obtained through future pre-clinical and clinical studies. Although this study has been performed using a stent retriever only technique, this can be expanded to combination therapies employing aspiration as well. Prior study Weafer et al. (2019) has demonstrated variation in stent indentation with composition of clot. Stent indentation obtained from radiopaque markers' position in this model can be used to determine the type of clot upon deploying the stent retriever. If an operator employs combination technique (Requena et al., 2023), an aspiration catheter can be introduced and tracked up to the stent. As discussed by Lee (2023), visualization of clot can further help with limiting fragmentation during retrieval. Although our current model demonstrates feasibility of clot boundary prediction in a single frame with a small dataset, this exact prediction can be applied to subsequent frames during retrieval which could help with clot retention and subsequently improve first pass efficacy. Puntonet et al. (2019) highlights the importance of clot retention by stating that infarcts in initially unaffected territories were reported in 1–8.6% of patients. They also emphasize that this is likely underestimated since most studies relied on follow up CT for this estimate.

Currently, for imaging of occlusive thrombi in AIS, methodologies such as high-resolution magnetic resonance imaging (HR-MRI), multimodal computed tomography (CT), ultrasonography and contrast-based imaging such as angiography are being explored (Zhang et al., 2023; Gasparian et al., 2015). Zhang et al. (2023) used 3D T1-weighted HR-MRI to identify intracranial thrombus. This can be useful in determining clot location and burden. However, may not provide characteristics of clot composition (Gasparian et al., 2015). CT is helpful in determining clot length accurately and non-contrast CT can be performed relatively quickly (Gasparian et al., 2015). Intraoperative contrast-based imaging techniques such as catheter angiography can be sensitive and offer *in-situ* treatment. It also helps define the location of proximal occlusions and corresponding collaterals (Gasparian et al., 2015). The predictive model described in this work offers the benefit of modeling the clot boundary and composition intraoperatively and can be used with catheter angiography that is currently employed in MT. It also offers the advantage of being incorporated within the current workflow, thereby not requiring additional procedural steps from the operator.

A potential challenge for the methodology presented in the current work is misplacement of the stent retriever by the operator. Imaging techniques such as contrast-enhanced cone beam CT (CE-CBCT) has been proposed by Hofmeister et al. (2025) to visualize the distant segment (or “dark side”) of the clot which could be beneficial. This study utilized standard CE-CBCT and 3D Rotational Angiography (3DRA) acquisition protocols predefined by the manufacturer and are commercially available, thereby aiding implementation. However, this was a single center, retrospective study with a limited sample size. Therefore, further work is needed to understand the workflow implications.

Another potential limitation is that the current version of this model is trained with limited data heterogeneity. This includes the use of a single commercial stent retriever, lack of variation with anatomy, clot type and retrieval technique. Furthermore, operator errors and use of different fluoroscopic equipment could also contribute to the performance of this model. Therefore, future training dataset obtained from pre-clinical and clinical studies must account for this variability to ensure that the model is exposed to these factors to provide better prediction.

## Conclusion

Our model accurately segments the clot from the original image in the anteroposterior (AP) view, with an average validation mean 2-class mean intersection over union (IOU) of 85.8% and area under the receiver operating characteristics (AUROC) of 89.9%. In future work, we plan to test these results in pre-clinical models to further refine the efficacy of these predictive models. Design changes to position and density of radiopaque markers on the stent can help refine the accuracy of prediction. Furthermore, this can lead to real-time 3-D reconstructions of clot engagement with the stent during retrieval which could further enhance first pass efficacy in endovascular thrombectomy procedures.

## Data Availability

The datasets presented in this article are not readily available because data used in this work is owned by Medtronic PLC. Requests to access the datasets should be directed to varun.kashyap@medtronic.com.
