# Supplementary material for: Training a high accuracy model to visualize blood clots during mechanical thrombectomy for the treatment of Acute Ischemic Stroke
Source: Front Stroke. 2025 Oct 17;4:1610399. doi: 10.3389/fstro.2025.1610399 (PMC12802791; doi:10.3389/fstro.2025.1610399)
Supplement: Supplementary file 1 [file Presentation_1.pdf]

Supplementary Information

TRAINING A HIGH ACCURACY MODEL TO VISUALIZE BLOOD CLOTS DURING MECHANICAL THROMBECTOMY FOR THE TREATMENT OF ACUTE ISCHEMIC STROKE

Varun Kashyap, Ph.D.*, Richard Zhu, M.S.+, Karthik Narasimhan, Ph.D.+

Affiliations

*Research and Technologies, Medtronic Neurovascular, Irvine, CA, USA

+Department of Computer Science, Princeton University, Princeton, NJ, USA

1. U-NET ARCHITECTURE

UNet architecture and implementation in Keras made available below, where img size is (450, 450).

```
inputs = keras.Input(shape=img_size + (1,))
# Contraction path
c1 = layers.Conv2D(16, (7, 7), activation='relu', kernel_initializer='
he_normal', padding='same')(inputs)
c1 = layers.Dropout(0.1)(c1)
c1 = layers.Conv2D(16, (7, 7), activation='relu', kernel_initializer='
he_normal', padding='same')(c1)
p1 = layers.MaxPooling2D((2, 2))(c1)
c2 = layers.Conv2D(32, (7, 7), activation='relu', kernel_initializer='
he_normal', padding='same')(p1)
c2 = layers.Dropout(0.1)(c2)
c2 = layers.Conv2D(32, (7, 7), activation='relu', kernel_initializer='
he_normal', padding='same')(c2)
p2 = layers.MaxPooling2D((2, 2))(c2)
# Bottleneck
c3 = layers.Conv2D(64, (7, 7), activation='relu', kernel_initializer='
he_normal', padding='same')(p2)
c3 = layers.Dropout(0.2)(c3)
c3 = layers.Conv2D(64, (7, 7), activation='relu', kernel_initializer='
he_normal', padding='same')(c3)
# Expansion path
u4 = layers.Conv2DTranspose(32, (2, 2), strides=(2, 2), padding='same')(
```

```
c3)
u4 = layers.concatenate([u4, c2])
c4 = layers.Conv2D(32, (7, 7), activation='relu', kernel_initializer='
he_normal', padding='same')(u4)
c4 = layers.Dropout(0.1)(c4)
c4 = layers.Conv2D(32, (7, 7), activation='relu', kernel_initializer='
he_normal', padding='same')(c4)
u5 = layers.Conv2DTranspose(16, (2, 2), strides=(2, 2), padding='same')(
c4)
u5 = layers.concatenate([u5, c1], axis=3)
c5 = layers.Conv2D(16, (7, 7), activation='relu', kernel_initializer='
he_normal', padding='same')(u5)
c5 = layers.Dropout(0.1)(c5)
c5 = layers.Conv2D(16, (7, 7), activation='relu', kernel_initializer='
he_normal', padding='same')(c5)
outputs = layers.Conv2D(1, (1, 1), activation='sigmoid')(c5)
model = keras.Model(inputs=[inputs], outputs=[outputs])
```

2. Additional Model Outputs

We investigated model performance on a held-out test set of data collected at the same time as our training/validation data (clot visible due to injection of radiopaque fluid). As shown in Figure S1, the predictions aligns closely, both visually and according to Mean IOU/AUROC scores, with the reference images. The second through fourth columns each correspond to a crop of the centermost 450x450 area of the original image.

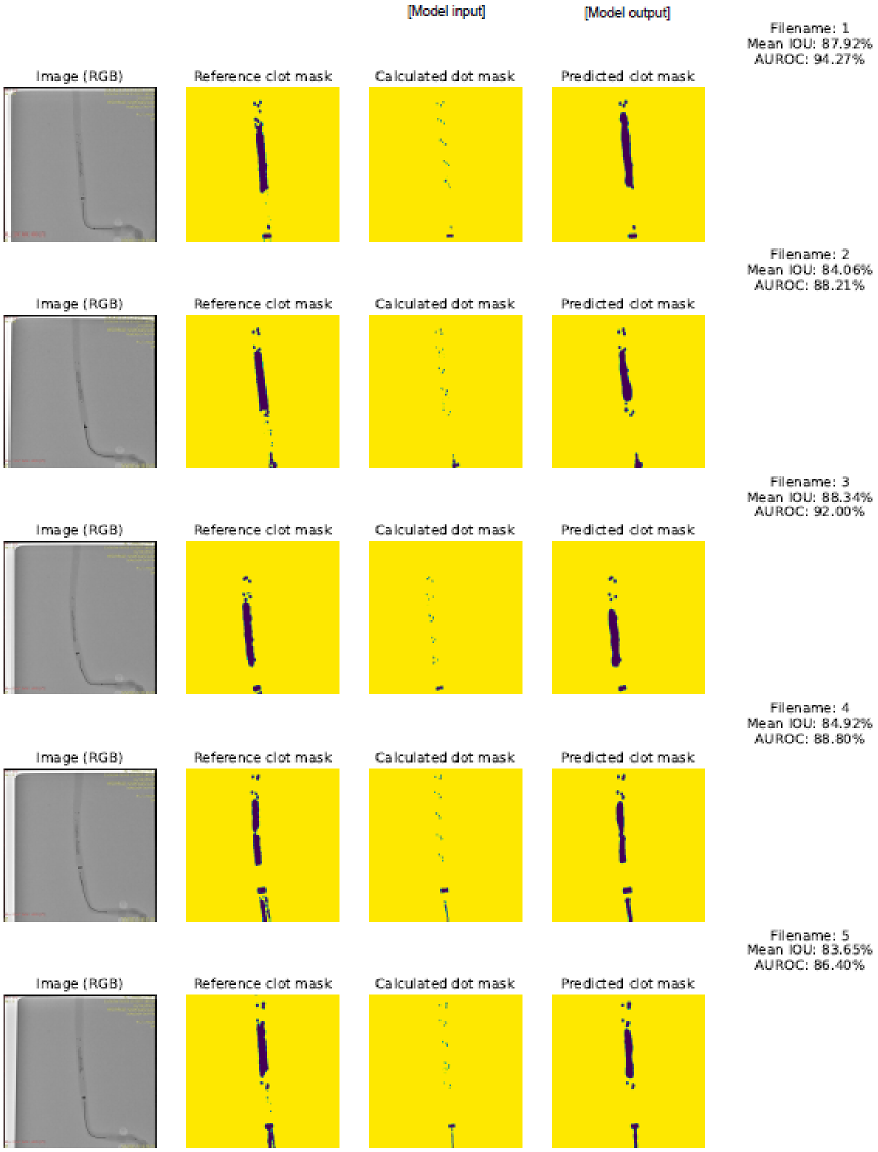

Figure S1: Performance on held-out test set. The first column ("Image (RGB)") represents the image taken by the device. The second column ("Reference clot mask") shows the clot mask extracted from the image, using grayscale thresholding; this is used as the ground truth during model training and evaluation. The third column ("Calculated dot mask") represents a mask containing only the

radiopaque markers, also extracted using grayscale thresholding; this is used as the model input. The fourth column (“Predicted clot mask”) is the mask of the clot generated by the model; the model output

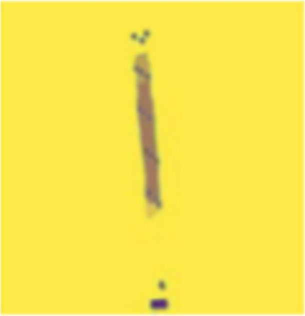

Figure S2: A visual overlay of the input, output, and reference images in Figure 4 of the manuscript, showing a close match in the direction normal to the clot but weaker match lengthwise. Specifically, the edges of input and output images align closely with the left- and right-most markers, while lengthwise this alignment is less evident.

We next investigate model performance on a second test set collected at a later date (with different frame size) to test the robustness of our model (Figure S3). The clots in this setup are not visible, since radiopaque fluid is not used, so we take optical images to visually compare our prediction accuracy. Qualitatively, we observe close correlation between prediction and reference when we align the optical image of the reference clot with the predicted clot mask based on the location of markers.

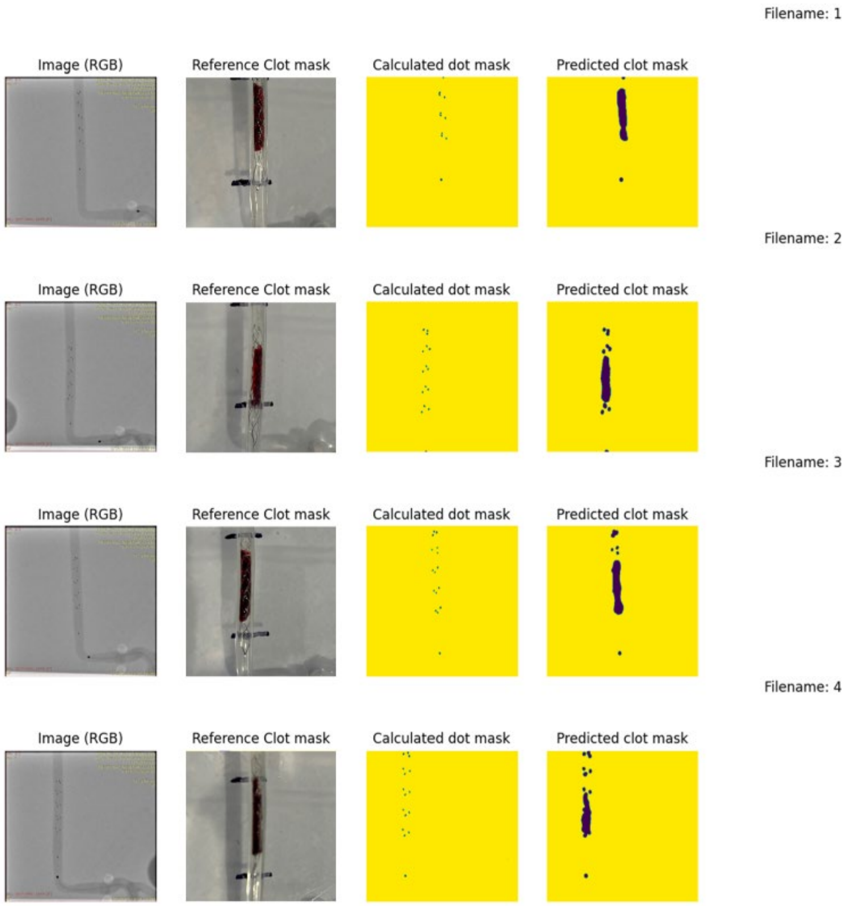

Figure S3: Results from our predictions using ClotNet on all images in the test set with an associated optical image (4 of 5). The model only sees the third column ('Calculated dot mask') which is programmatically extracted from the first column ('Image (RGB)')
